# Artificial intelligence for chest radiography: an overview of techniques, challenges, and future directions

**DOI:** 10.1038/s44401-026-00087-y

**Published:** 2026-06-02

**Authors:** Hidetoshi Matsuo, Mizuho Nishio, Koji Fujimoto, Nicolas Deperrois, Takaaki Matsunaga, Farhad Nooralahzadeh, Michael Krauthammer, Takamichi Murakami

**Affiliations:** 1 Department of Radiology, Kobe University, Kobe, Japan; 2 Department of Advanced Imaging in Medical Magnetic Resonance, Kyoto University, Kyoto, Japan; 3 Department of Quantitative Biomedicine, University of Zurich, Zurich, Switzerland

**Keywords:** Computational biology and bioinformatics, Engineering, Health care, Mathematics and computing, Medical research

## Abstract

This paper presents a critical analysis of AI advancements in chest radiograph (CXR) analysis, tracing its evolution from conventional machine learning to deep learning and multimodal approaches. Early models relied on hand-crafted features, while recent CNNs and transformer-based architectures now achieve diagnostic accuracies exceeding or comparable to radiologists for various thoracic conditions. The recent integration of large language models and multimodal systems—combining imaging with clinical text—has further improved performance and interpretability. Despite notable success, challenges still remain, including model bias, limited generalisation across institutions, and explainability. Solutions such as data sharing, domain adaptation, and explainable-AI techniques are actively being explored. Looking forward, AI systems trained on diverse patient data streams promise enhanced clinical integration and diagnostic precision.

## Introduction

### Chest X-Ray and the Emergence of AI

**Fig. 1:**
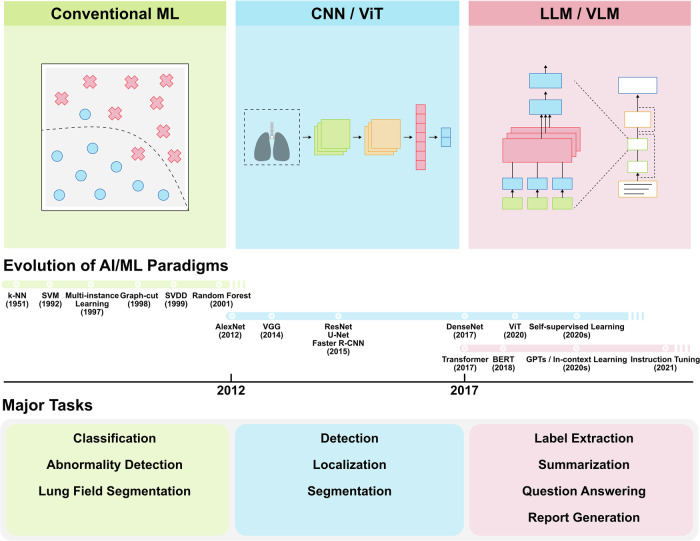
Overview of AI approaches in chest radiography. The figure shows the evolution from traditional machine learning (ML) methods using handcrafted features to convolutional neural networks (CNNs) with end-to-end learning, and the recent emergence of large language models (LLMs) for multimodal analysis of chest X-rays. The central timeline marks representative milestones (e.g., AlexNet, Transformer/BERT, ViT, self‑supervised learning, and instruction tuning), with 2012 and 2017 indicating inflection points for deep learning and transformer‑based methods. The bottom row maps dominant tasks to each era—(left) classification/abnormality detection/lung‑field segmentation, (middle) detection/localisation/segmentation, and (right) text‑centric tasks such as label extraction, summarisation, question answering, and report generation. Abbreviations: ViT, Vision Transformer; VLM, Vision‑Language Model. This schematic is illustrative rather than exhaustive; dates are approximate.

**Fig. 2:**
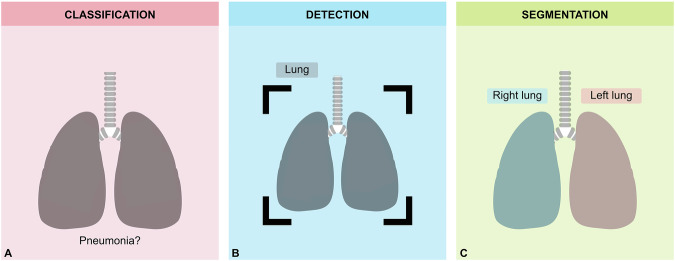
Three primary AI tasks in chest radiography analysis. **A** Classification: determining the presence of diseases such as pneumonia from chest X-ray images. **B** Detection: localising abnormalities within lung regions using bounding boxes or attention mechanisms. **C** Segmentation: precise pixel-level delineation of anatomical structures including left and right lung boundaries.

**Fig. 3:**
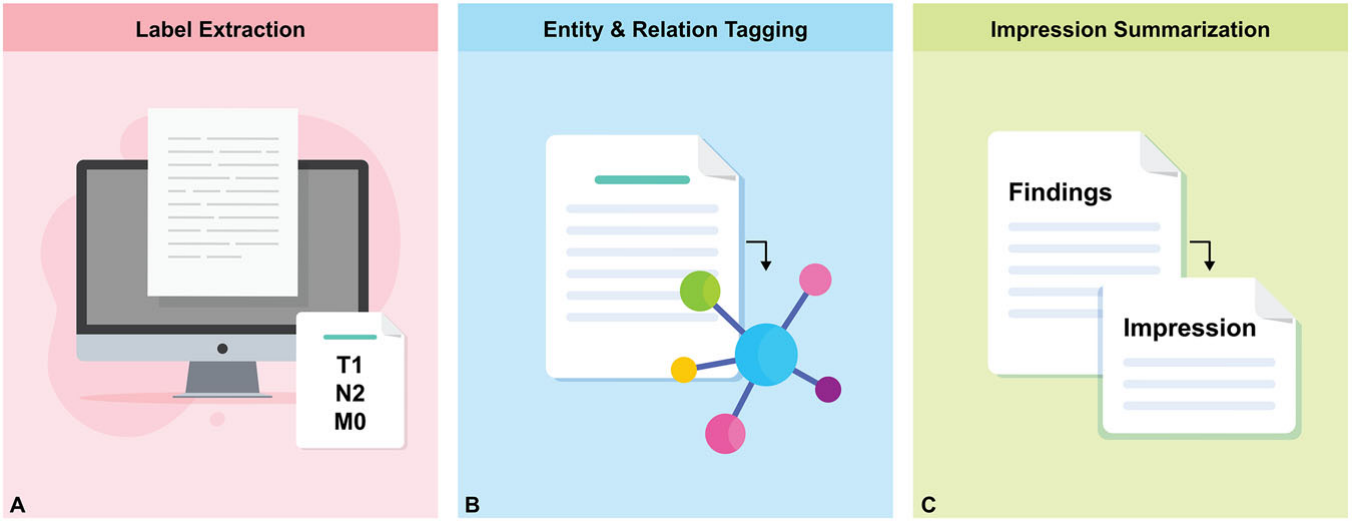
AI applications in radiology report processing. **A** Label extraction: automated extraction of structured information such as TNM staging from radiology reports. **B** Entity and relation tagging: identification and relationship mapping of medical entities within report text. **C** Impression summarisation: automated generation of concise clinical impressions from detailed radiological findings.

Chest radiograph (CXR) is a fundamental imaging examination routinely used for diagnosing chest diseases such as pneumonia and tuberculosis^1^. However, interpreting CXR images requires specialised knowledge and experience, and there have been concerns about the risk of missed or misdiagnosed lesions^2,3^. The recent rise of deep learning has led to remarkable advancements in the automation and sophistication of CXR analysis, with AI models achieving expert level performance. An overview of the evolution of these AI approaches in chest radiography is presented in Fig. 1. Fig. 2 illustrates three primary AI tasks in chest radiography analysis, and Fig. 3 outlines three primary AI applications in radiology report processing. Table 1 summarises the main representative AI model families used for the chest X-ray analysis. These advancements have been supported by the emergence of large-scale annotated datasets and advanced neural network architectures. Thus, AI is expected to serve as a tool that alleviates the workload of radiologists and promotes more standardised diagnoses.

**Table 1:** Representative AI model families used for chest X‑ray analysis and their key characteristics, spanning classical machine‑learning methods, convolutional neural networks, transformer‑based language models, and multimodal vision–language models

Model	Category	Key idea / approach
SVM (Support Vector Machine)	Classical ML	Discriminative model that maximizes the margin between classes; with appropriate kernels it can handle non-linear decision boundaries^54^.
RF (Random Forest)	Classical ML	Ensemble of decision trees; predicts by majority vote for classification and averaging for regression^55^.
k-NN (k-Nearest Neighbors)	Classical ML	Instance-based classifier that assigns labels based on the majority label among the k nearest neighbours in feature space^56^.
Logistic Regression	Classical ML	Linear model that applies a sigmoid to a weighted feature sum and interprets the output as the posterior probability of the positive class^57^.
CheXNet	CNN	DenseNet-121 trained on the NIH ChestX-ray14 dataset for multi-label chest X-ray abnormality classification^25^.
AlexNet	CNN	Early deep CNN with 5 convolutional and 3 fully connected layers, popularizing large-scale image classification^73^.
VGG network	CNN	Deep CNN architecture that stacks many small 3×3 convolution filters (e.g. VGG-16/19)^74^.
ResNet (Residual Network)	CNN	CNN with residual (skip) connections that enable residual learning and stable training of very deep networks^75^.
DenseNet	CNN	CNN where each layer is connected to all subsequent layers within a block, promoting feature reuse and parameter efficiency^76^.
U-Net	CNN (segmentation)	Encoder–decoder CNN with skip connections, designed for medical image segmentation to preserve context while recovering precise spatial detail^86^.
EfficientNet	CNN	CNN family that jointly scales network depth, width and input resolution using a compound scaling coefficient^82^.
ViT (Vision Transformer)	Transformer-based	Vision Transformer that splits an image into patches, embeds them as tokens, and applies a pure Transformer encoder for image recognition^78^.
CheXzero	Self-supervised vision–language	CLIP-style contrastive pretraining on CXR–report image–text pairs, enabling zero-shot multi-label chest pathology classification^79^.
BERT	Pretrained language model (Transformer encoder)	Bidirectional Transformer encoder pretrained on large general-domain corpora with masked language modelling and next-sentence prediction^154^.
CheXbert	BERT-based radiology NLP	BERT-based automatic labeler that extracts 14 chest findings from free-text chest X-ray reports^155^.
DeBERTa	BERT-derivative encoder	BERT-style encoder with disentangled content and position embeddings and a decoding-enhanced masked language modelling objective^156^.
GPT (Generative Pre-trained Transformer) family	Autoregressive LLM	Family of autoregressive Transformer LLMs for general-purpose text generation and in-context few-shot learning^157,158^.
CheX-GPT	Hybrid LLM	Uses GPT-4 to generate pseudo-labels for chest X-ray reports and trains a BERT-based report labeler (CheX-GPT) purely on these LLM-derived labels^107^.
T5 (Text-to-Text Transfer Transformer)	Encoder–decoder LLM	Treats all NLP tasks in a unified text-to-text format using an encoder–decoder Transformer^159^.
Med-PaLM M	Proprietary multimodal medical foundation model	Google’s medical foundation model that uses a single set of weights to handle clinical text, medical images and genomics within one multimodal architecture^160^.
Med-Gemini	Proprietary multimodal medical foundation model	Gemini-based family of medical multimodal models (2D/3D medical imaging and other modalities)^127^.
LLaVA-Med	Public (research only) medical multimodal VLM	Medical VLM instruction-tuned for image–text dialogue using PMC figures and captions with GPT-4-generated self-instruct data and curriculum learning^128^.
CheXagent	Public (research only) multimodal VLM	CXR-focused multimodal VLM instruction-tuned on the instruct dataset built from public chest X-ray datasets, supporting tasks such as report generation and finding enumeration^133^.
RoentGen	Text-conditioned diffusion model	Domain-adapted latent diffusion model that generates realistic CXR images conditioned on radiology reports or other textual prompts^97^.
RadVLM	Public (research only) multimodal VLM	Compact CXR-specific conversational VLM that unifies report draft generation, presence/absence listing of 14 findings, bounding-box grounding and multi-turn dialogue in a single model^12^.

*CLIP* contrastive language-image pre-training, *ResNEt* residual network, *GPT* generative pretrained transformer, *ViT* Vision Transformer, *NLP* natural language processing, *LLM* large language model, *SVM* support vector machine, *RF* random forest, *k-NN* k-Nearest neighbors, *CNN* convolutional neural network, *VLM* vision–language model, *BERT* bidirectional encoder representations from transformers.

The performance of AI-based CXR analysis has improved in recent years, even exceeding the diagnostic accuracy of physicians for certain findings. For instance, convolutional neural networks have outperformed radiologists in detecting atelectasis^4^, and sensitivity and specificity to other lesions have continued to improve thanks to refined models. Recent developments in 2024–2025 have introduced vision-language models that can generate detailed radiological reports^5,6^, foundation models pre-trained on large-scale medical datasets, and transformer-based architectures that demonstrate superior performance across multiple pathological findings^5,7,8^. Thus, the trajectory of AI-based CXR diagnostic support has progressed steadily from research to practical use, showing important advancements not only in accuracy but also in clinical utility.

Clinical implementation of AI-based CXR analysis has shown promising developments alongside persistent challenges, with notable successes. In one trial using an AI-based triage system in a real clinical setting, the average turnaround time for CXR reports decreased dramatically from 11.2 days to 2.7 days, indicating that automated prioritisation by AI greatly improved workflow efficiency^5^. Furthermore, some AI solutions for CXR are now entering practical use in various countries; for example, in 2019, the U.S. Food and Drug Administration (FDA) approved an algorithm for the rapid detection of pneumothorax on portable CXR, marking a significant milestone^6^.

Despite the potential of deep learning–based CXR analysis systems and these early successes, their adoption in clinical practice remains limited, and the rate of dissemination has not been as rapid as initially anticipated^9^. Several issues related to the reliability and generalisability of AI models underlie this situation. First, building a highly accurate model requires large amounts of high-quality data, yet the collection and sharing of medical data pose challenges related to privacy and standardisation. Second, the ‘black box’ nature of deep learning can make the reasoning behind model outputs difficult to interpret, creating a barrier to gaining the trust of healthcare providers. Furthermore, integrating AI into existing hospital systems (e.g., PACS and report-generation workflows) poses both technical and operational difficulties^10^. Rigorous validation across multiple facilities and regions, as well as the development of explainable AI (XAI), is critical for demonstrating the true utility of deep learning models and enhancing their trustworthiness.

Looking forward, two major trajectories stand out: multimodal AI and Large Language Models (LLMs). Multimodal AI integrates not only image data but also clinical information from electronic medical records (e.g., patient history and laboratory results) and other test findings, enabling a more comprehensive approach that goes beyond what can be gleaned from imaging data alone^11^. AI-driven diagnostic support that incorporates patient history and clinical findings is also expected to become increasingly prevalent in CXR analysis^12^.

This accelerating progress is now inseparable from advances in multimodal AI for CXR analysis. State-of-the-art multimodal architectures typically pair an image encoder with an LLM, enabling joint reasoning over pixel information and diverse clinical context (e.g., patient history, laboratory data, and free-text reports). Improvements in LLM capacity and in-context learning therefore translate directly into richer vision-language models that can both interpret the radiograph and situate its findings within the broader clinical picture.

In addition to powering full image–text systems, LLM components are already being applied to text-centric tasks such as automated report drafting and interactive question answering. For example, recent studies show that conditioning an LLM on the findings section of a radiology report allows it to generate a coherent impression paragraph, illustrating how language priors can complement image-based reasoning rather than replace it. Together, these developments suggest a continuum: stronger LLMs expand the expressive and integrative capabilities of multimodal CXR models, while multimodal CXR datasets in turn provide valuable test beds for probing and improving medical-domain LLM performance.

This review provides a comprehensive overview of AI in the context of CXR, covering CXR datasets, traditional machine learning, convolutional neural networks, large language models, and multimodal AI. We detail the current advancements in CXR-related AI, discuss the challenges, and explore future perspectives for its practical implementation and continued development.

## Dataset of CXR

### Current status of CXR image datasets

CXR imaging remains one of the most frequently performed diagnostic examinations in clinical medicine, and the vast archives of these images have recently spurred extensive deep-learning research. To accelerate AI development, the research community has released a number of large-scale public datasets. However, many studies still rely on private datasets accumulated within hospitals or companies, making comparison with public data essential for reproducibility and benchmarking. This section reviews the representative CXR datasets, grouping them by task—classification, detection, segmentation, and report generation—and summarising for each dataset its scale, data-collection method, annotation scheme, and availability. This section is intended to clarify both the significance and the challenges of these datasets for clinical specialists and computer-science researchers alike.

### Overview of public datasets

Several public CXR repositories now comprise hundreds of thousands of images. A prominent example is ChestX-ray14, released by the U.S. NIH, which contains 112,120 frontal radiographs drawn from 30,805 patients and annotated with fourteen disease labels^13^. In this dataset, each label was assigned automatically by extracting keywords from the accompanying radiology report; consequently, common thoracic conditions such as pneumonia, pulmonary nodules, and pneumothorax are all represented. Introduced in 2017 as the first hospital-scale open CXR collection, ChestX-ray14 quickly became a baseline for numerous studies; nevertheless, later work showed that the automatically mined labels include noise and uncertainty that can impair model performance^14^.

Seeking to address that limitation, Stanford University released CheXpert^15^, a much larger dataset comprising 224,316 radiographs from 65,240 patients imaged at Stanford Hospital between 2002 and 2017. Fourteen findings were automatically extracted from each report and attached to every image as three-way labels—positive, negative, or uncertain—while a rule-based labeller was provided to allow researchers to decide how to handle uncertain cases during training. Published in 2019, CheXpert also introduced a 500-study test set that had been manually annotated by radiologists, thereby enabling direct comparison of AI performance to that of humans; this dataset was launched in a competition format to encourage standardised benchmarking.

Around the same time, an even larger corpus derived from the Beth Israel Deaconess Medical Center PACS: 377,110 radiographs paired with 227,835 full-text reports and representing more than 65,000 patients, was made available by the MIMIC-CXR project^16^. Although MIMIC-CXR does not supply image-level labels, investigators can mine their own findings from the reports or exploit the image–text pairs for automated report-generation research. Distributed under the broader MIMIC license—access requires completion of a data-use training course—the collection has rapidly become a cornerstone for multi-task learning and large-scale vision–language modelling.

In Europe, a Valencian research group released PadChest in 2020^17^, comprising more than 160,000 high-resolution CXRs and their Spanish-language reports collected from 67,000 patients between 2009 and 2017. Each report has been tagged with 174 radiographic findings, 19 differential diagnoses and 104 anatomical locations; roughly 27% of the tags were assigned manually by radiologists, with the remainder supplied by an attention-based RNN and subsequently verified. PadChest therefore offers unusually rich, multilingual annotations for multi-label classification, and its six projection views (PA/AP, lateral, etc.) add further diversity. More recently, the same group has developed PadChest-GR, which augments the original dataset with bounding-box annotations, enabling localisation tasks alongside the existing classification capabilities^18^. Researchers can obtain access by applying through the official website^19^.

Two frequently used resources serve different purposes from the hospital‑scale datasets above. Indiana University (Open‑I) links 7470 CXRs to 3955 English‑language reports, providing a compact, report‑centric corpus that is widely used in language‑focused studies^20^. MIDRC, a multi‑institutional data commons launched during the COVID‑19 pandemic, offers both open and sequestered tiers; its curated RICORD CXR subset includes 998 radiologist‑annotated images from 361 patients using a standardised labelling schema and is well-suited for COVID‑focused classification and generalisation studies^21,22^.

Alongside these large-scale resources, several historically important small datasets remain in use. The Japan Radiological Society’s JSRT database, created in 2000, contains 247 images—154 with pulmonary nodules of varying difficulty and 93 normal controls—together with presence/absence labels^23^. To support tuberculosis research, the NIH released the Montgomery County dataset (138 images: 58 TB, 80 normal) and the Shenzhen dataset (662 images: 336 TB, 326 normal) in 2014^24^; each radiograph carries a TB label and brief findings, and lung masks are provided for the Montgomery images. Although far smaller than today’s mega-datasets, such targeted collections continue to underpin disease-specific detection and classification studies.

### Datasets for classification tasks

For tasks that determine the presence of abnormalities or classify diseases in CXR images, the large-scale public collections described above are most frequently used. NIH ChestX-ray14 (ChestX-ray14)^13^ and CheXpert^15^ have become standard benchmarks for multi-label classification, where a single image may be associated with multiple disease tags. The CheXNet model introduced by Rajpurkar and colleagues, for example, reported pneumonia detection performance on ChestX-ray14 that outperformed radiologists (AUC 0.768 vs 0.71) and surpassed earlier machine-learning methods across the dataset’s fourteen-disease task average^25^. Consequently, ChestX-ray14 is now the de-facto reference for comparing deep-learning architectures in thoracic disease classification. However, its automatically generated labels contain noise; therefore, further gains depend on label clean-up and handling uncertainty^26,27^. CheXpert mitigates this issue by detecting negation and uncertainty expressions in the reports and introducing an ‘uncertain’ class, giving modellers explicit control over how such cases are treated^15^. This refinement promises more reliable classification benchmarks.

Beyond these multi-label datasets, specialised two-class resources exist. The tuberculosis collections from Montgomery County and Shenzhen, mentioned earlier, are routinely used to evaluate ‘TB vs. normal’ detectors; by augmenting and merging them, researchers often reach several thousand training images^24^. For paediatric pneumonia, a Kaggle set released by Guangzhou Medical Center provides 5,863 paediatric CXRs labelled as pneumonia or normal, enabling early deep-learning studies^28^. Because paediatric findings differ from adult disease patterns, models trained solely on adult data transfer poorly to children. For COVID-19-specific classification, the MIDRC-RICORD CXR release provides radiologist‑verified diagnostic labels for 998 images collected across four international sites, enabling standardised evaluation and cross‑site generalisation analyses^22^.

To close this gap, large paediatric CXR datasets have begun to appear. In 2023, Nguyen and colleagues introduced PediCXR (VinDr-PCXR)^29,30^, the first large-scale collection purpose-built for detecting paediatric thoracic diseases. The dataset comprises 9125 CXRs from patients aged 10 years or younger, acquired at a Vietnamese children’s hospital. Radiologists with more than ten years of experience annotated 36 findings and 15 diagnoses, adding bounding boxes around each abnormality. Divided into 7728 training images and 1397 test images, PediCXR supplies detailed lesion-level supervision and is expected to accelerate research on conditions such as paediatric pneumonia, bronchitis, and congenital heart disease.

### Datasets for detection and localisation tasks

Accurately locating abnormal findings on a chest radiograph requires datasets with high-quality spatial annotations. The earlier large collections—ChestX-ray14 and CheXpert—provide only image-level labels and therefore cannot answer the clinically crucial question of *where* a lesion is situated^31^. To address this, several publicly available datasets now include radiologist-drawn bounding boxes or masks.

A leading example is VinDr-CXR^31^, which is drawn from more than 100,000 hospital radiographs. The release contains 18,000 frontal images that 17 radiologists annotated in detail. Each study includes 22 local findings and six global diagnoses, with every abnormality marked by a bounding-box. For the 15,000 training images, three radiologists independently labelled each case; for the 3000-image test set, five experts reached a consensus label. Owing to this rigorous procedure, VinDr-CXR offers the largest set of high-quality expert annotations for public lesion-detection research and supports both pure detection and combined ‘classification + localisation’ tasks aimed at improving diagnostic accuracy and model interpretability.

Complementing the VinDr‑CXR, the paediatric PediCXR dataset likewise provides bounding‑box annotations for its 9125 images, enabling localisation studies in young patients and bridging the adult–child domain gap^29,30^. By contrast, the RICORD CXR release supplies image‑level expert labels rather than bounding boxes or masks, and is therefore used primarily for classification or severity scoring rather than lesion localisation^21^.

In the United States, the RSNA Pneumonia Detection Challenge of 2018^32^ supplied bounding-box annotations for pneumonia-suspicious opacities on 26,684 images extracted from ChestX-ray14^13^. Although the task evaluated both binary classification (pneumonia vs. normal) and lesion localisation, it chiefly popularised the use of generic object-detection frameworks such as Faster R-CNN in medical imaging.

Several small yet precisely annotated datasets pre-date these efforts. The JSRT database includes not only nodule presence but also exact nodule contours, supporting early studies of pulmonary-nodule detection^23^.

### Datasets for segmentation tasks

Segmentation in chest radiography involves extracting specific organs or lesion regions at the pixel level. Compared with classification or detection, public resources remain scarce, yet several initiatives deserve mention. For anatomical structures, an international group created the *Segmentation in Chest Radiographs* (SCR) dataset by adding lung-field and cardiac silhouettes to the JSRT images; it is now a standard benchmark for cross-method accuracy comparison^33^. Likewise, the original Montgomery County tuberculosis collection supplies expert lung masks for each of its 138 radiographs, enabling analyses of lesion topography relative to lung fields^24^. In addition, a merged set of 704 lung masks—drawn from the Montgomery County (138 images) and Shenzhen (566 images) tuberculosis collections—is freely available on Kaggle and widely used^24^.

For lesion segmentation, the flagship public resource is the 2019 SIIM–ACR Pneumothorax Segmentation dataset, released for a crowdsourcing challenge. All 12,047 radiographs are accompanied by radiologist-delineated masks that outline the extent of pneumothorax, with 3,205 images held out for testing^34^. The competition demonstrated the effectiveness of U-Net–style architectures and catalysed the adoption of segmentation techniques in clinical AI. During the COVID-19 pandemic, many studies sought to segment pulmonary infiltrates on CXR, prompting the emergency release of small but valuable datasets that include opacity masks—for example, the COVID-19 Image Data Collection^35^ and BIMCV-COVID + ^36^. Nonetheless, the inherently diffuse boundaries of some thoracic lesions make pixel-level annotation labour-intensive; consequently, the volume of available public data remains limited.

Recently, semi-automated pipelines have produced much larger resources. Chest ImaGenome adds bounding boxes for 29 anatomical regions to 242k MIMIC-CXR images^37,38^. CXLSeg offers 243k automatically generated lung masks^39^, while CheXmask aggregates fine-grained masks across multiple institutions to yield 650k segmented CXRs^40^. These coarse-mask datasets, when combined with smaller sets of meticulously hand-drawn labels, are fuelling research on mixed-supervision strategies and are expected to drive further gains in segmentation performance. In addition, Chest ImaGenome also supplies structured scene‑graph relations among the 29 anatomical regions, which enable spatial reasoning and longitudinal comparison for grounding and change‑detection tasks^37,38^. Likewise, CheXmask harmonises more than 650k fine‑grained anatomical masks across ChestX‑ray14, CheXpert, MIMIC‑CXR‑JPG, PadChest, and VinDr‑CXR, enabling scalable training of anatomical and lesion‑aware models and facilitating mixed‑supervision pipelines^40^.

### Datasets for report-generation tasks

Automatic report generation, that is, producing full narrative findings and impressions directly from a chest radiograph, requires paired image-text data. Conceptually, the task resembles medical image captioning; hence, the number of image–report pairs is a critical factor. Among public resources, MIMIC-CXR^16^ remains the most frequently used and one of the largest among public resources. Because it couples hundreds of thousands of images with their complete radiology reports, researchers can train deep-learning models that generate richly detailed prose. More recently, CheXpert Plus has been released as another large-scale paired CXR–report resource, providing 223,462 report–radiograph pairs across 187,711 studies. In parallel, CXPMRG-Bench benchmarks various report-generation models and large-model baselines on CheXpert Plus, facilitating standardised comparisons^41,42^. Indeed, since 2019, numerous RNN- and Transformer-based systems have been trained on MIMIC-CXR in an effort to improve clinical accuracy and textual coherence^43–45^.

Before MIMIC, the main source of paired data was the NIH’s Open-I (Indiana University Chest X-ray Collection)^46^. This archive contains 7470 images linked to 3955 English-language reports and was originally designed as a searchable repository for medical-journal figures. Described in detail by Demner-Fushman and colleagues in 2016, Open-I long remained the sole public image–report dataset, but its modest size and predominance of normal studies limited the diversity of abnormal descriptions that models could learn^41,47^. With the advent of MIMIC-CXR, report generation became feasible at a clinically realistic scale, and state-of-the-art models are now almost always evaluated on this corpus^6^ and, more recently, on CheXpert Plus via standardized benchmarks such as CXPMRG-Bench. Figs. 2, 3.

Although PadChest^17^ also provides paired images and Spanish-language reports, it is more frequently used for multi-label finding classification than for free-text generation^48,49^. Nevertheless, its linguistic diversity makes it a valuable complement when exploring multilingual report-generation models. Table 2 summarises the main public CXR datasets discussed above, including their scale, primary tasks, and available annotations.

**Table 2:** Representative public chest X‑ray (CXR) datasets used in deep‑learning research, summarising for each resource its provider, approximate scale (number of images and patients), primary target tasks (e.g. classification, detection, segmentation, report generation), and available labels or annotations

Dataset	Provider/dataset	No. of images/patients	Main task(s)	Labels/annotations
Chest X-ray14	NIH	112,120 images/30,805 patients	Classification	14 disease labels automatically extracted from reports (noisy labels).
CheXpert	Stanford University	224,316 images/65,240 patients	Classification	14 finding labels automatically extracted; three-way labels (positive/negative/uncertain). Includes a manually annotated test set of 500 studies.
MIMIC-CXR	Beth Israel Deaconess Medical Center	377,110 images/ > 65,000 patients	Report generation, multi-task learning	Paired with 227,835 full-text radiology reports.
BIMCV; PadChest / PadChest-GR	Medical Image Bank of the Valencian Community	>160,000 images/67,000 patients	Multi-label classification, multilingual report generation, detection	174 radiographic findings, 19 differential diagnoses and 104 anatomical locations; ~27% of labels manually annotated.
JSRT	Japanese Society of Radiological Technology	247 images (154 nodule, 93 normal)	Pulmonary-nodule detection	Nodule presence/absence and benign/malignant label; nodule centre coordinates and size.
Montgomery County	NIH	138 images (58 TB, 80 normal)	Tuberculosis detection, lung-field segmentation	TB label, brief findings, lung masks.
Shenzhen	NIH	662 images (336 TB, 326 normal)	Tuberculosis detection	TB labels and brief findings.
PediCXR / VinDr-PCXR	Nguyen et al.	9125 images/patients ≤ 10 years old	Paediatric chest-disease detection and localisation	36 findings and 15 diagnoses annotated by radiologists; bounding boxes for abnormal regions.
VinDr-CXR	Nguyen et al.	18,000 images	Detection and localisation; classification + localisation	22 findings and 6 diagnoses; bounding boxes for abnormalities.
RSNA Pneumonia Detection Challenge	RSNA	26,684 images (subset of ChestX-ray14)	Pneumonia detection and localisation	Bounding boxes for pneumonia-suspicious opacities.
SCR: Segmentation in Chest Radiographs	International group	247 images (JSRT images with masks)	Anatomical-structure segmentation	Segmentation masks for lung fields, heart and clavicles.
SIIM–ACR Pneumothorax Segmentation	SIIM–ACR	12,047 images	Pneumothorax segmentation	Radiologist-delineated masks indicating pneumothorax extent.
COVID-19 Image Data Collection	Cohen et al.	761 images/412 patients (as of Sept 2020; expanding)	Multiple tasks (detection, severity estimation, prognosis prediction)	Clinical outcome metadata; severity scores for a subset of cases.
BIMCV-COVID+	BIMCV	2265 images/1311 patients (initial release; expanding)	Classification, detection, linkage with reports	UMLS-mapped finding labels and anatomical labels; paired reports and examination metadata.
Chest ImaGenome	Wu et al.	242,072 images (CXRs derived from MIMIC-CXR)	Extraction of anatomical bounding boxes; scene-graph structuring	Bounding boxes for 29 anatomical regions; 1,256 relation types; >670,000 temporal comparison relations.
CXLSeg	Nimalsiri et al.	243,324 images (CXRs derived from MIMIC-CXR)	Lung-mask segmentation	Automatically generated lung masks.
CheXmask	Gaggion et al.	657,566 images (from five public CXR datasets)	Anatomical segmentation	Harmonised lung and heart masks for five public CXR datasets.
Open-I	Indiana University	7470 images/3955 reports	Report generation	English-language reports paired with images.

### Caveat on biases in public CXR datasets

The use of public CXR datasets entails a risk of systematic bias arising from confounding and limited labels/metadata. A systematic review of COVID‑19 CXR resources documented the prevalence of poorly documented ‘remix’ datasets and cross‑source class mixing, which can cause models to leverage site‑ or device‑specific cues and thus inflate performance estimates. These issues are not unique to COVID‑19 and should be kept in mind when interpreting benchmarks built on public data^50^.

### Challenges surrounding private datasets and data sharing

Despite the recent wave of large public CXR releases, the vast majority of chest radiographs stored in hospitals worldwide remain inaccessible within private datasets. The volume of images acquired in daily clinical practice dwarfs what any public corpus offers, and many research groups or companies assemble proprietary archives that greatly exceed public sets in scale. Anderson et al. exemplify this trend: they aggregated 341,355 CXR examinations—nearly 490,000 images—from 15 U.S. hospitals and clinics for model training and evaluated its performance against another 20,000 unseen private studies, achieving excellent generalisation, with its performance comparable to that of expert readers^51^. Models trained on such large, multi-institutional private data often prove highly robust, yet their performance is difficult for outsiders to reproduce or verify because contractual and privacy constraints prevent data release. Consequently, research papers frequently cite ‘institutional datasets’ or ‘multi-site private cohorts’ without providing access.

This opacity hampers reproducibility and hinders fair assessment of how well results will transfer beyond the original institutions; several reports document substantial domain shifts between private and public data^52,53^. In other words, an AI system that excels on open benchmarks may not perform equally well in everyday clinical settings.

To address the gap, future public datasets must better approximate real-world case distributions and include high-fidelity annotations. Projects such as VinDr-CXR and PediCXR illustrate this approach: expert labelling combined with multi-centre image collection increases both quality and diversity. Another practical strategy is transfer learning, which involves pre-training a model on several large public corpora, then fine-tuning it on smaller target datasets, whether public or private.

Ethical and legal considerations also remain paramount. Most open CXR resources are free for research use only, and many (e.g., CheXpert, MIMIC-CXR) require users to remove re-identification information and complete data-use agreements^15^. Private datasets likewise demand local ethics-board approval, de-identification, and, in many jurisdictions, informed consent^31^. As international data sharing expands, researchers will need frameworks that comply with regulations such as HIPAA and GDPR while enabling scientific progress. Notably, MIDRC demonstrates a pragmatic two‑tier model—open and sequestered commons—coupled with a live explorer and community annotation workflows, which may inform future governance of large CXR repositories^22^.

In summary, this survey has outlined the principal CXR datasets available today. The richness of large, publicly accessible collections now permits objective comparison and steady gains in model performance across classification, detection, segmentation, and report-generation tasks. At the same time, label noise, sampling bias, and distribution gaps between public and private data remain unresolved. Continued expansion and refinement of open datasets, coupled with evaluation protocols that mirror clinical reality, will be essential for building the evidence base needed to translate CXR AI into routine practice.

## Conventional machine learning for CXR

### Overview of conventional machine learning algorithms

Traditional computer vision approaches often rely on manually engineered features for classification or prediction, using classical machine learning algorithms. This section provides an overview of representative conventional machine learning methods applied to CXR analysis, focusing particularly on support vector machines (SVM), random forests (RF), k-nearest neighbours (k-NN), and logistic regression.

#### Support vector machine (SVM)

SVM is a discriminative model based on margin maximisation, classifying data in a high-dimensional feature space^54^. By choosing an appropriate kernel function, it can handle nonlinear separability. SVMs have been widely adopted in medical image classification, especially for small-to-medium-sized datasets, owing to their robust classification performance.

#### Random forest (RF)

RF is an ensemble learning method based on multiple decision trees^55^. It builds each decision tree with random subsets of the training data and aggregates their predictions by majority voting. RF is less prone to overfitting and can provide estimates of feature importance, making it useful for feature-based classification of radiological images. It has been successfully applied to tasks such as texture- or shape-based disease classification.

#### k-Nearest neighbours (k-NN)

k-NN is a simple non-parametric method that classifies new samples based on distances to the ‘neighbouring’ samples in the feature space^56^. Although it can be computationally costly and may suffer from the curse of dimensionality, it is easy to implement and often used as a baseline method. In CXR analysis, k-NN has been used for abnormality scoring and classification tasks in small-scale datasets.

#### Logistic regression

Logistic regression is a linear model that uses the sigmoid function, allowing its outputs to be interpreted as probabilities^57^. While it assumes a linear relationship between features and the target class, it has relatively few parameters and is thus more interpretable. It has been applied in medical imaging for disease risk estimation and for combining outputs from other models (i.e., serving as a meta-classifier in ensemble methods).

These algorithms were predominant in CXR analysis before the emergence of deep learning, and each was utilised based on its unique characteristics.

## Main tasks in CXR analysis and examples of conventional approaches

Conventional machine learning methods have been applied to a variety of tasks for CXR, including classification, abnormality detection, and lesion segmentation. This section introduces typical approaches for each task in existing studies. Deep learning–based methods will be covered in subsequent chapters; therefore, they are not addressed here.

### Image classification

Image-level classification, determining the presence or absence of findings or classifying disease types from an entire CXR, is a quintessential application of traditional methods. Generally, regions of interest (ROIs; e.g., lung fields or lung apices) are first extracted, followed by the computation of hand-engineered features such as histograms, texture descriptors, or shape characteristics. These features are then fed into the aforementioned classifiers (SVM, RF, etc.) to distinguish normal vs. abnormal or to categorise disease.

### Tuberculosis screening

In tuberculosis screening, machine learning approaches often involve extracting texture features from lung fields and using various algorithms to classify normal vs. TB cases. Some studies have reported high accuracy with these methods^58^. However, performance can vary depending on the choice of features and datasets. In a comparative study that included simple neural networks, SVM, k-NN, and RF, accuracy for TB detection was approximately 80% in some cases^59^.

### Pneumonia classification

Studies have also employed texture-based features to classify paediatric pneumonia or COVID-19 pneumonia on CXR. Methods such as k-NN, SVM, and RF have been used to build models^60^. Leveraging features such as radial intensity distributions, fractal dimensions, or superpixel-based descriptors, some studies have reported accuracies of 89–91% for paediatric pneumonia and 95–99% for COVID-19 pneumonia. In some cases, these carefully engineered features combined with conventional classifiers can achieve performance comparable to that of deep learning.

### Multi-class classification

Traditional approaches have also been applied to multi-class classification tasks, where different diseases or abnormal regions are identified within the same CXR. For instance, on the ImageCLEFmed2005 dataset with 15 categories, the use of shape and texture features selected by a genetic algorithm and then classified by RF or SVM yielded an accuracy of up to 89%. Another study combined wavelet transforms with local binary patterns (LBP) and employed RF for classifying X-ray images into 30 categories, achieving 93.1% precision and 89.4% recall^61^. Although these examples were not limited to CXRs, they demonstrate the effectiveness of feature engineering and ensemble learning for multi-class tasks.

### Abnormality detection (Outlier detection)

Abnormality detection aims to automatically identify findings that deviate from norm. Although this can be viewed as a classification task, one unique challenge is that, in practice, the set of possible abnormalities may not be fully represented during training. One-class classification methods are often used when abnormal data are insufficient. A typical example is a one-class SVM or related methods such as Support Vector Data Description (SVDD), which train only on normal images and label images that deviate substantially from this distribution as ‘abnormal’^62^. In CXR applications, the model quantifies how closely a new image fits within the normal pattern; if the score exceeds a certain threshold, the image is deemed abnormal. However, these methods often entail high computational costs and can be sensitive to the choice of features, limiting their scalability and consistency^63^.

Some supervised approaches attempt local detection of abnormalities. For example, multi-instance learning (MIL) can be used, where the CXR is divided into multiple patches, each assigned an abnormality score. In one study, MIL was applied to detect TB lesions, achieving a per-pixel AUC of approximately 0.87^58,64^. While such methods can locate lesion regions based on weak labels (e.g., ‘image-level’ labels only), their accuracy remains limited compared to modern techniques.

### Lesion segmentation

Segmentation in CXR focuses on delineating specific structures or lesion areas in the image at the pixel level. Traditionally, the primary target has been the extraction of the lung fields (lung segmentation), which is crucial for stabilising subsequent steps such as abnormality detection and feature extraction. Classical segmentation techniques include thresholding, region growing, active shape/active contour methods, and graph cuts. Candemir et al. proposed a graph-cut-based method that starts from an average lung shape model and refines the lung boundary via graph cuts^65^. While this approach yields accurate lung segmentation, it struggles to accommodate large variations in lung shape and may require several seconds per 1024×1024 CXR, posing challenges for large-scale processing.

Pixel-wise classification using conventional machine learning has also been employed for segmentation. Each pixel is characterised by local intensity or texture features, and a traditional classifier is then used to decide whether it belongs to the lung field, heart shadow, or background^66^. Such methods, based on shallow classifiers, can yield reasonable masks for lungs or the heart. Before the advent of deep learning, pixel-based classification or rule-based segmentation methods were standard practices^67^.

In contrast, segmenting the lesion itself (e.g., pneumonia shadows or TB foci) in CXR is particularly difficult owing to the overlapping nature of 3D structures and the ambiguity of lesion boundaries. Additionally, obtaining pixel-level annotations can be challenging. Consequently, prior to deep learning, explicit lesion segmentation methods were relatively rare in CXR; lesion localisation was more often performed by detecting candidate regions and classifying them rather than generating precise segmentation masks. Thus, most conventional machine learning segmentation efforts have been limited to extracting anatomical regions such as the lungs or organ silhouettes.

### Challenges in conventional methods

Despite their widespread use, several challenges have been identified in applying traditional machine learning approaches to CXR analysis:

#### Feature engineering burden

Compared with deep learning (discussed later), conventional methods require extensive labour to design and extract suitable image features. Substantial effort is needed for image preprocessing and region-of-interest extraction, and multiple feature types must be tested and optimised for different models^68,69^. This manual engineering process depends heavily on domain expertise, is not easily generalised, and often must be redone for each new disease or image type.

#### Limitations in accuracy

Hand-engineered features combined with shallow classifiers may fail to fully leverage the rich information in complex images, leading to performance plateaus. As datasets grow larger and more diverse, the accuracy of conventional methods often stagnates. In the eve of the deep learning era, these performance limitations are becoming more apparent.

#### Lack of explainability

While explainability is crucial for clinical applications, most traditional algorithms are not fully transparent. Although random forests can output feature importance, their final decisions are aggregated across many trees, making explanations less intuitive for radiologists. With SVM and k-NN, it can be even harder to explain why a particular image is classified as abnormal. If the features themselves are interpretable, some partial explanations are possible; yet, it remains difficult to pinpoint which specific region of the image influenced the decision. This lack of interpretability remains an obstacle to building trust in AI-based diagnostic support tools.

### Computational cost and scalability

The conventional pipeline typically involves multiple steps—preprocessing (e.g., segmentation), feature extraction, and classifier application—each consuming computational resources and time. Some feature extraction methods can take several seconds per image, which becomes problematic for large-scale or real-time processing^70^. Additionally, the scalability of certain algorithms is limited; for instance, the computational and memory requirements of a nonlinear SVM increase sharply with the size of the training data, and k-NN requires distance computations against the entire training set at inference time^71^. These factors restrict the size and complexity of datasets that conventional methods can handle efficiently.

Because of these limitations, conventional machine learning approaches alone have struggled to meet the increasing demands for accuracy, versatility, and efficiency in CXR analysis. This situation set the stage for the emergence of deep learning methods, which automate feature learning and often provide superior performance (discussed in the next chapter). Nevertheless, the insights from traditional approaches continue to inform current practices, especially in preprocessing and interpretability efforts, underscoring the importance of understanding both the strengths and weaknesses of classical methods.

## Deep learning models for CXR image analysis

### Overview of deep learning and its application to chest radiographs

Deep learning has revolutionised image analysis technologies, with rapid expansion into the field of medical imaging. Among various modalities, CXRs are one of the most frequently used imaging examinations in clinical practice^72^. Since the late 2010s, researchers have aimed to develop deep learning models that assist or automate the detection of thoracic abnormalities,which was previously performed by human readers^15^.

A notable example is CheXNet^13^, a deep learning model based on a 121-layer convolutional neural network (CNN) (DenseNet-121) trained on the ChestX-ray14 dataset constructed by Wang et al. CheXNet demonstrated performance exceeding that of average radiologists in pneumonia detection based on the F1 score^25^. Subsequently, the same group released CheXpert, a large-scale dataset comprising over 100,000 CXRs with labels extracted from corresponding radiology reports, addressing issues related to label accuracy and uncertainty management^15^. These initiatives have sparked significant interest in the application of deep learning for chest radiograph interpretation in the medical domain.

### Major Deep Learning Architectures for Image Analysis

Various deep learning architectures have been employed for image analysis. CNNs, which extract hierarchical features from images, have notably improved image recognition accuracy, as demonstrated by models such as AlexNet^73^ and the VGG network^74^. The VGG network achieved high accuracy in the ILSVRC 2014 classification task by stacking small 3 × 3 convolutional filters to construct networks up to 19 layers deep^74^.

The introduction of the Residual Network (ResNet) incorporated a ‘residual learning’ framework that enabled the successful training of extremely deep networks, reaching up to 152 layers^75^. ResNet alleviates the vanishing gradient problem by learning residual mappings through skip connections, thereby enhancing the training efficiency and performance of deep networks^75^. DenseNet^76^ further evolved this approach by connecting each layer to every subsequent layer, promoting feature reuse and parameter efficiency.

These CNN-based architectures have been widely applied to chest radiograph analysis. For instance, the CheXNet mentioned earlier, is based on DenseNet-121, with modifications to the final fully connected layer to classify pneumonia using the ChestX-ray14 dataset^25^.

More recently, transformer-based models such as the Vision Transformer (ViT) have been introduced into image domains. ViT applies the Transformer architecture originally developed for natural language processing^77^ by splitting images into patches treated as tokens. Although ViT models generally require large-scale pre-training because of reduced inductive biases compared to CNNs, they can outperform CNNs given sufficient data^78^.

In the field of chest radiograph analysis, Tiu et al. developed a multi-modal model based on ViT that performs self-supervised pre-training using only CXRs and radiology reports. The model achieved performance comparable to that of radiologists with minimal labelled data and without pre-training on ImageNet^79^.

## Representative tasks in chest radiograph analysis

### Classification (Image-level classification)

A major task in CXR analysis is image-level classification, where the presence or absence of specific diseases such as pneumonia or tuberculosis is determined based on the entire image^13^. While CheXNet exemplifies this approach, other studies have explored the classification of tuberculosis^80^ and COVID-19 infections^81^ using networks such as ResNet and EfficientNet^82^.

### Detection and localisation (Lesion detection)

Detecting and localising specific abnormalities within CXRs involves^83^ identifying lesions such as nodules, masses, or pneumothorax using bounding boxes. This task can be formulated as an object-detection problem.

Weakly supervised learning approaches have been proposed to infer lesion locations from image-level labels alone, with techniques such as Class Activation Mapping (CAM) enabling localisation by highlighting discriminative regions within CNNs^84^. While Grad-CAM has been widely used for lesion localisation in CXRs, hierarchical attention models^85^ have demonstrated improved localisation accuracy by integrating anatomical priors and enabling joint learning of localisation and diagnosis from image-level labels. Meanwhile, the VinDr-CXR dataset^31^, comprising 18,000 chest radiographs with bounding-box annotations for 22 abnormal findings, has enabled supervised training and objective benchmarking. For example, Pham et al. reported a mean AUROC of 0.967 and a localisation sensitivity of 80.2% at 1.0 false-positive lesions per scan (FROC) for their explainable VinDr-CXR CAD system, which combines an EfficientNet-B6 classifier and an EfficientDet-D6 detector, under their internal training and evaluation protocol^31^. Therefore, these values are system- and protocol-specific rather than representative of all models trained on the public VinDr-CXR release.

### Segmentation (Region extraction)

Semantic segmentation involves extracting ROIs at the pixel level. The introduction of U-Net^86^ marked a milestone in medical image segmentation, leading to robust methods for lung field segmentation. EfficientNet-based architectures have further enhanced the precision and automation of lung segmentation tasks^87^.

### Generalisation and data bias

Deep learning models are heavily dependent on the statistical properties of the training data, and thus, performance often degrades when tested on data from different institutions or patient populations. Zech et al. (2018) demonstrated that models trained on data from one hospital showed reduced performance on external datasets^52^. Avoiding spurious correlations and improving cross-institutional generalisability remain critical challenges.

### Explainability

A major concern in medical applications is the lack of model interpretability. Techniques such as Grad-CAM^88^ attempt to visualise regions of focus within the image, yet complete elucidation of the decision-making process of deep models remains elusive. Comprehensive reviews of XAI in healthcare emphasise that interpretability is essential for clinical deployment^89^. For example, in an external-validation reader study on COVID-19 chest radiographs, Miyazaki et al. used Grad-CAM to interrogate model decisions alongside radiologist assessments (with and without AI assistance)^90^. Heat-maps in correctly classified cases were concentrated within anatomically plausible lung regions corresponding to peripheral opacities, whereas failure cases revealed attention on spurious cues (e.g., device/marker regions or pleural margins); this provides concrete, case-level insight into when explanations align with clinical reasoning and when they do not. This qualitative audit illustrates how visualisation can substantiate claims about model success and diagnose failure modes during real-world deployment.

### Computational and data limitations

The high resolution of CXRs imposes considerable computational demands for processing and analysis. Moreover, obtaining high-quality annotated datasets remains a bottleneck, with rare diseases often underrepresented, causing class imbalances that negatively affect model performance^1^.

To address these challenges, self-supervised learning frameworks based on contrastive learning, such as CheXzero^79^, have emerged. CheXzero employs a CLIP-like architecture to learn joint representations from image–text pairs (chest X-rays and associated reports), enabling disease classification and zero-shot inference without the need for extensive manual annotations. Fine-tuning with small amounts of labelled data enabled the model to achieve a performance comparable to that of radiologists.

## Synthetic data generation and augmentation for CXR

### Overview

To address class imbalance, privacy constraints, and the scarcity of rare findings, recent studies synthesise chest radiographs (CXRs) and mix them with real data for model training^91^. Classical GAN‑based approaches can produce lesion‑conditioned or class‑targeted images^92–94^, whereas newer diffusion, including latent‑diffusion, models deliver higher‑fidelity textures and controllable edits^5,95,96^. Beyond unconditional synthesis, instruction‑ or report‑conditioned generators leverage paired image–text corpora to align images with clinical descriptions. RoentGen is a representative report‑conditioned CXR generator^97^, and foundation‑level VLMs tailored to CXR have demonstrated realistic synthesis and image–text alignment^79,98^.

### Generative architectures and conditioning

Generative pipelines span GANs (adversarial training), DDPMs (denoising diffusion), and latent‑diffusion (generation in a compressed latent space)^95,96^. Conditioning strategies include class/lesion conditioning, text/report conditioning, and mask‑guided inpainting, enabling controllable edits and study‑specific variations^97,99,100^. In data‑scarce regimes, diffusion‑based augmentation has been reported to support downstream classification/segmentation when mixed judiciously with real data, while early CXR‑focused GAN studies showed mixed yet often positive effects for pneumonia/TB classification^93,94,97,101,102^.

### Representative applications

Controlled augmentation can increase sensitivity for rare targets (e.g., tension pneumothorax, device malposition), support hard‑negative mining, and stress‑test robustness under distribution shifts (view position, exposure, simulated devices)^97,99^. When paired with small sets of radiologist‑drawn masks, copy‑paste or in‑painting pipelines can cheaply expand lesion‑level supervision (weak‑to‑strong label upgrading)^103–105^. In practice, teams often (i) pretrain on large image–text corpora, (ii) introduce targeted synthetic samples for under‑represented findings, and (iii) validate strictly on fully real external test sets to quantify net benefit^5,91^.

### Generalisation and bias

Synthetic images can embed spurious cues (textures, borders, sampling artifacts) that inflate internal metrics yet degrade external validity. Effects may vary across institutions and subgroups; therefore stratified external evaluation is required to detect domain‑shift amplification rather than mitigation^79,91^.

### Quality control, privacy, and safeguards

Generative models potentially memorise or leak training data, which raises significant privacy concerns that have been documented in the literature. To address these documented risks while maintaining model quality, researchers have proposed various quality‑assurance and safety protocols^106^. Several key practices have been proposed in the literature for addressing these challenges: first, recording provenance tags for every sample in the training manifest, indicating whether it is real or synthetic and specifying the generator, version, and conditioning used; second, before mixing synthetic with real images, conducting blinded realism checks by radiologists focused on anatomy, devices, and pathology plausibility; third, including stratified external testing on fully real datasets, with subgroup analyses to detect distribution‑ or demographic‑specific failures; fourth, running ablation studies that vary the synthetic‑to‑real ratio and the conditioning strategy (e.g., unconditional versus report‑conditioned) to quantify the sensitivity to mixing policies; and finally, performing privacy audits, such as membership‑inference and memorisation probes, and applying conservative data‑curation practices to reduce leakage risk^5^.

### Practical guidance and reporting

Synthetic data can be used to balance or stress-test rather than to replace clinical diversity. Furthermore, the generation pipeline should be reported in full—model family, conditioning signals, and prompt templates, together with target proportions, mixing ratios, and performance gains measured on external real-world test sets. Evidence from recent CXR image-generation and CXR-specific foundation/VLM work indicates that improvements hinge primarily on careful mixing policies rather than on synthetic scale alone^5,91^.

## Large language models for chest X-ray

### Single modal large language models in general model

In recent years, the application of LLMs to medical text has gained momentum in the field of CXR. Rather than multimodal models that process imaging data directly, LLMs that take only text, such as radiology reports and clinical notes, as input are demonstrating sophisticated language processing capabilities grounded in clinical knowledge learned from large-scale report corpora. This section provides an overview of the latest research trends in text-based LLMs that do not rely on pixel-level image input in the CXR domain. For text-only pipelines in CXR, two resources now anchor auditable supervision: GPT-assisted labelling frameworks (e.g., CheX-GPT) and large expert-annotated entity–relation corpora (e.g., RadGraph-XL). The former improves label quality over rule-based systems, while the latter benchmarks information extraction and clinical consistency; together they complement vision–language efforts by supplying robust, auditable text supervision^107,108^.

### Automatic labelling and information extraction from radiology reports

Extracting labels for diseases and findings from vast collections of radiology reports associated with CXR datasets is a critical foundation for training diagnostic AI models. However, conventional rule-based labellers, which rely on grammar parsing and keyword matching, are often cumbersome to design and adjust and may not perform reliably. Studies have reported that the accuracy of such rule-based labels—for example, in the ChestXray14 dataset—was significantly lower than initially reported^27,109^.

Transformer-based models have significantly advanced this field. Smit et al. fine-tuned a BERT model pre-trained on biomedical texts to develop ‘CheXbert,’ which extracted 14 chest findings from CheXpert reports with high accuracy. CheXbert significantly outperformed rule-based methods and has become a new benchmark for report labelling^110^. Tejani et al. later evaluated various BERT-derived models for identifying the presence of chest lines and devices, showing that newer Transformer models such as DeBERTa achieved AUROCs ranging from 0.845 to 0.925 even with a small number of labelled reports, outperforming classical BERT^111^. These models also enhanced the detection of negations, for instance, more accurately identifying ‘removal of a tracheal tube’ as a negated condition compared to earlier methods^109^.

More recently, generative LLMs such as those based on the GPT family have been applied to this task. Abdullah et al. fine-tuned a GPT-based model for report labelling using the MIMIC-CXR dataset and reported a mean F1 score of 0.901 across 14 metrics, surpassing the rule-based CheXpert labeller (0.886) and approaching CheXbert’s performance (0.905), with no statistically significant difference between GPT and CheXbert, but a significant advantage over CheXpert^15^. A hybrid approach called ‘CheX-GPT,’ which leverages GPT-4 for label generation and uses the resulting data to train a BERT-based classifier, has recently shown performance exceeding that of CheXbert by combining GPT’s contextual comprehension with the efficiency of BERT architecture^107^.

Beyond label extraction, efforts have been made to structure free-text reports. The RadGraph project proposed a schema for tagging entities (e.g., Anatomy, Observation) and their relations in CXR reports, leading to the creation of large-scale datasets: RadGraph-1.0 (600 reports) and RadGraph-XL (2,300 reports)^108,112^. RadGraph-XL includes over 410,000 annotations across four modalities, including chest radiographs. Interestingly, fine-tuned BERT models significantly outperformed GPT-4 used in zero- or few-shot settings, achieving a relation extraction F1 score of 0.69 compared to approximately 0.02 for GPT-4. This finding underscores the necessity of domain-specific fine-tuning, even for large-scale general-purpose LLMs^108^.

Efforts have also emerged to extract findings (e.g., pneumothorax, pleural effusion) from CXR reports using open-source LLMs without fine-tuning, operating in local environments. Nowak et al. demonstrated that some of these LLMs, when prompted in zero-shot settings, performed comparably to GPT-4o, suggesting that on-premise LLM applications can be privacy-conscious and feasible in healthcare^113^.

Beyond rule‑based and BERT‑style classifiers, recent work has compared commercial and open‑source LLMs for labelling CXR reports. Dorfner et al. evaluated GPT‑4 against large open‑source models from the Llama, Qwen, and Mixtral families across public and institutional datasets, including ImaGenome. GPT‑4 was superior in zero‑shot settings, but few‑shot prompting brought the strongest open‑source systems to near‑parity, highlighting a privacy‑preserving on‑premises option when cloud use is constrained^114^.

### Automated summarisation of CXR reports

Automatically generating the ‘Impression’ section from the ‘findings’ of radiology reports represents a clinically significant application. The impression condenses key findings and conveys diagnostic conclusions but is highly dependent on individual physician styles, making automation challenging. Recently, large generative models have been increasingly adopted to tackle this issue. However, reported performance can differ substantially depending on whether models are fine-tuned on in-domain reports or used via zero-shot prompting, and fair comparison requires that prompt conditions (e.g., instruction template, output-format constraints, few-shot examples, and decoding settings such as temperature) be clearly specified.

Ziegelmayer et al. used GPT-4 to generate impressions from findings-only input and had radiologists assess the quality. In a study of 25 cases, GPT-4-generated impressions were rated comparable in quality to human-written ones. Although human summaries scored marginally higher in coherence, accuracy, and completeness, the differences were not statistically significant. Notably, radiologists struggled to distinguish between human and AI-generated impressions, with a misidentification rate of approximately 39% under text-only conditions^115^. Sun et al. reported that while GPT-4 impressions were occasionally less detailed, referring clinicians sometimes found them easier to understand than those written by radiologists^116^. Nishio et al. trained a T5 (Text-to-Text Transformer) model for CXR report summarisation, achieving a ROUGE-L score of approximately 54 and receiving favorable clinical utility ratings in 85% of cases^117^. Generally, fine-tuning can better capture domain-specific terminology and institution-specific reporting style and tends to improve faithfulness and completeness; however, zero-shot prompting may underperform for strict extraction/labelling or rare findings due to limited task-specific calibration and high sensitivity to prompt formulation and decoding settings.

These findings suggest that AI-generated summaries may be nearly indistinguishable from those written by experts. However, the absence of standardised metrics for evaluating clinical validity, such as factual consistency between findings and impressions, omission of critical/actionable findings, and the presence of high-risk errors that could change management, remains a limitation^116^. Effective deployment of such models requires human oversight, consistency-check algorithms, or feedback mechanisms from specialists.　In recent years, automatic metrics that enable objective evaluation of clinical validity, such as RadEval, the radiology-specific COMET metric proposed by Calamida et al.^118^., have been advancing, and a robust quantitative evaluation infrastructure is now eagerly anticipated.

Advanced strategies are also being explored. These include retrieve-and-generate systems that enhance summaries by referencing similar cases from a report database and multi-agent systems that pair LLMs with separate verification agents to detect and correct inconsistencies^119^. As models grow in capability, it is anticipated that such techniques will support more standardised and efficient impression generation, contributing to improved report quality and productivity.

### Multimodal large language models in general model

A new trend in LLM research is the development of multimodal models that incorporate vision capabilities, enabling them to process images and respond to multimodal instructions. Proprietary systems, such as GPT-4o^120^, Gemini^121^, or Claude^122^ demonstrate strong general-purpose vision–language performance. In parallel, open-source VLMs (e.g., LLaVA^123^, Qwen2-VL^124^, and InternVL^125^) have reported competitive results on several public multimodal benchmarks (e.g., MMBench, MMMU, and DocVQA), which cover tasks such as general visual question answering and instruction following, multimodal reasoning, and document/OCR-style understanding. Performance on these benchmarks is commonly reported using metrics such as multiple-choice accuracy, exact match, or averaged benchmark scores (e.g., an ‘overall score’ aggregated across subsets). Importantly, cross-model comparisons are highly benchmark- and protocol-dependent (e.g., prompt format, image resolution, tool use, and model version), and proprietary models may still lead to broader or more challenging reasoning suites. Therefore, rather than claiming universal parity, we describe open-source models as competitive on selected benchmarks under comparable evaluation settings rather than claiming universal parity. The training principle behind these models consists of building an instruction-following dataset that contains user-assistant question and answers (Q&A) and dialogues, each paired with one or several images, with the Q&As reflecting the image’s content^123^. Notably, strong performance on general-domain benchmarks does not necessarily translate to medical imaging tasks, where domain shift, safety requirements, and clinically grounded evaluation criteria are critical.

### Vision and language models applied to chest X-ray

The success of VLMs in the general domain inspired the development of medical-based VLMs, particularly where image-based interpretation is critical. In terms of proprietary models, Med-PaLM^126^ and Med-Gemini^127^ showed impressive performance across a range of multimodal medical tasks, including VQA, report generation, and summarisation. In parallel, open-source models based on the same training approach as LLaVA, such as LLaVA-Med^128^, were developed using biomedical datasets from PubMed to design instructions and multi-turn conversations paired with medical images. Among these medical applications, CXR interpretation is a key area of interest. While early AI-driven models primarily focused on single-task report generation^129–132^, more recent work consists of building multimodal, foundation CXR models that can perform multiple tasks, such as classification, detection, grounding, or image generation. Notable examples include CheXagent^133^, which aggregates multiple CXR datasets into a unified instruction-tuning corpus and demonstrates strong multi-task capability across settings such as classification, grounding, and report generation, compared with prior task-specific baselines. Meanwhile, RoentGen^97^ showed capabilities in generating CXR images in addition to generating CXR reports. Other approaches, such as Wolf^134^, RaDialog^135^, and M4CXR^136^ incorporate conversational abilities, while the recent RadVLM^12^ combines multiple tasks within a single conversational thread.

Recent clinical reader and accuracy studies have begun to quantify the real-world value of domain-specific multimodal generative models that produce preliminary CXR reports. In a multicentre accuracy study, Hong et al. trained a domain-specific VLM on a large, multi-institutional corpus of radiographs and reports; the model showed high sensitivity for critical findings and outperformed a general-purpose GPT-4-Vision baseline^137^, with radiologists more often preferring and accepting the model-generated drafts. Complementing this, a reader study by the same group found that providing AI-generated preliminary reports shortened average reading time and improved agreement and perceived quality, while also increasing the sensitivity for several abnormalities; importantly, inter-reader variability persisted, underscoring the need for human oversight^138^. Taken together, these studies provide prospective evidence that, when used as an assistive tool, domain-specific multimodal generative AI can enhance CXR workflow efficiency and clinical reporting quality.

## Discussion and conclusion

### Overall summary

This article presents a comprehensive review of technological progress in CXR analysis, tracing the evolution from conventional machine-learning methods through deep learning to today’s multimodal AI approaches. Representative model families and their key characteristics across this spectrum—from classical ML algorithms to CNNs, transformer-based language models, and multimodal vision–language models—are summarised in Table 1. We first describe the transition from traditional pipelines—hand-crafted feature engineering coupled with shallow classifiers—to CNNs. Fuelled by large-scale datasets and increasing computational power, CNN-based systems now achieve markedly higher accuracy in the automatic detection and classification of lesions on CXR. Recent studies report diagnostic accuracies exceeding 90% for a wide range of diseases, including lung cancer, pneumonia, tuberculosis, and COVID-19^139^. These results demonstrate the potential of deep-learning tools to support radiologists and enhance clinical workflow.

We then highlighted the latest trend: applying LLMs and multimodal architectures to CXR interpretation. By fusing imaging data with clinical text—such as electronic health-record entries and radiology reports—multimodal AI systems have begun to deliver diagnostic performance superior to image-only models^140^. For instance, models that integrate CXR images with patient-level clinical parameters have achieved significantly higher AUCs than counterparts relying solely on images^140^. Parallel developments are yielding multimodal LLMs that ingest both images and text to generate structured findings or full radiology reports automatically^141^. Because these models emulate the radiologist’s diagnostic workflow—synthesising image features with clinical context—they have attracted growing attention; early experimental results already show report-generation quality approaching that of board-certified specialists.

In summary, AI for CXR analysis has progressed rapidly: evolving from conventional feature-based algorithms to deep learning and now toward multimodal AI that promises even greater diagnostic accuracy and interpretability.

## Challenges and solutions for clinical implementation

### Model bias

Even high-performing AI systems can inherit biases from imbalanced training data, leading to uneven diagnostic accuracy across patient subgroups. For example, Glocker et al. evaluated subgroup performance for disease detection on CheXpert (including the ‘No Finding’ and ‘Pleural Effusion’ labels) and reported subgroup true-positive rate (TPR)/false-positive rates (FPR) at a fixed operating point: the decision threshold was optimized per model to achieve FPR = 0.20 on the overall cohort, and performance was summarized using the Youden J statistic (J = TPR − FPR) at this target FPR. Under this protocol, relative performance on ‘No Finding’ decreased by 6.8–7.8% for female patients and performance on ‘Pleural Effusion’ decreased by 10.7–11.6% for Black patients, compared with average performance across subgroups^142^. Such disparities raise safety concerns in real-world use^143,144^. Mitigation strategies include assembling training cohorts that span diverse demographics and institutions, adding regularisation terms that discourage over-reliance on protected attributes, and quantifying inequity with fairness metrics such that models can be adjusted iteratively.

### Explainability

Because deep networks act as black boxes, clinicians must be able to understand why a system arrived at a particular conclusion before trusting it. XAI techniques such as Grad-CAM heat-maps^145^ and feature-attribution tools such as LIME are increasingly applied to medical images^146^. In CXR studies, visualising the regions that resulted in a pneumonia prediction helps physicians grasp the model’s rationale^146^. User studies report generally positive attitudes—explanations boost confidence; yet, many clinicians remain unfamiliar with XAI’s capabilities and limitations. Researchers therefore advocate multimodal explanations (e.g., linking highlighted image areas to textual clinical context) and dedicated training such that end-users can interpret outputs correctly, paving the way for smoother human-AI collaboration.

### Generalisation ability

A persistent hurdle is domain shift: performance often degrades when a model encounters data from new hospitals or patient populations whose scanners, acquisition protocols, or disease prevalence differ from the training set^52,147^. In CXR research, applying a model trained in one country to images from another can cause substantial accuracy losses^148^. Remedies include (i) data diversification: training on multi-institutional, multi-ethnic cohorts from the outset, and (ii) domain-adaptation techniques that fine-tune or recalibrate the network to the target distribution^147,149^. Supervised adaptation studies have already shown significant recovery of lost accuracy when models are retrained—even briefly—on representative external data. Future work should broaden validation to truly global cohorts and explore self-supervised or domain-invariant feature extractors that remain robust to unseen distributions^150^.

### Future perspectives

This final section outlines the anticipated directions for artificial-intelligence research and deployment in CXR analysis. Continued advances in model design, cross-fertilisation of ideas from other domains, wider adoption of multimodal architectures, and concerted efforts toward clinical integration are expected to accelerate progress.

### Ongoing evolution of AI techniques

As model architectures and training paradigms continue to mature, both the performance and the scope of CXR-focused AI are poised to expand. Transformer-based networks and self-supervised pre-training have recently emerged as powerful alternatives to conventional convolutional models, and foundation models jointly trained on images and text are beginning to appear in medical imaging^12,151^. Such models learn generalisable feature representations; therefore, they can be adapted to new tasks with only modest amounts of additional data, making high-accuracy, customised systems more attainable. Ongoing work on multimodal foundation models and large-scale unsupervised learning promises simultaneous improvements in accuracy, integration, and generalisation^152^. Ultimately, next-generation AI that learns from the full breadth of patient data will be central to precision medicine, supporting diagnosis and therapy tailored to the individual.

### Technology transfer from non-medical domains

Rapid progress in non-medical AI offers a rich source of innovation for healthcare. Examples include retraining LLMs developed for natural-language processing on medical corpora to handle guideline-based question answering and adapting sensor-fusion techniques from autonomous driving to integrate multiple imaging modalities. Similar cross-disciplinary transfers in CXR analysis could yield breakthroughs in performance and open new clinical applications.

### Expansion of multimodal AI

Future systems are likely to progress beyond single-modality analysis toward holistic models that integrate diverse data streams, such as additional imaging (CT, MRI), laboratory values, vital signs, medical history, genomic information and biomarkers. Such multimodal AI can emulate the way clinicians synthesise chart data with image findings, enabling early disease detection, refined severity grading, and more accurate outcome prediction. For example, when a subtle opacity is detected on CXR, a multimodal model could automatically generate a ranked differential diagnosis by combining imaging cues with the patient’s history and current symptoms. Institution-specific fine-tuning of shared multimodal foundation models may also help harmonise diagnostic quality across centres. Addressing complex medical problems that exceed the reach of single-source solutions will increasingly depend on this multimodal paradigm.

### Practical implementation in clinical workflows

Realising the full value of AI demands robust implementation within hospital IT infrastructure. Future work must focus on seamlessly integrating AI outputs into existing PACS and electronic health-record systems, supported by intuitive user-interface and user-experience design. Examples include unobtrusive alerts or predictive scores that appear automatically during image review, available for consultation whenever the physician needs them. Prospective clinical trials and pilot deployments will be critical for demonstrating tangible benefits—shorter reporting times, fewer missed findings and reduced inter-reader variability—thereby lowering adoption barriers. Evidence from simulated and real-world studies indicates that AI-assisted triage can markedly reduce reporting delays for critical CXR findings^153^. A sustainable operational model will also require mechanisms for continual learning, allowing models to incorporate feedback and adapt to evolving practice patterns or emerging diseases. Ultimately, optimal diagnostic performance should arise from synergistic collaboration between AI systems and healthcare professionals.

In summary, AI for CXR analysis has progressed rapidly from proof-of-concept accuracy studies toward practical clinical utility. Addressing the remaining challenges while maintaining close collaboration among researchers, clinicians, and industry will be essential for building trustworthy, high-impact AI systems. Such collective effort is expected to enhance diagnostic precision, strengthen healthcare delivery, and ultimately improve patient outcomes.

## Data Availability

All data generated or analysed during this study are included in this published article.
